# Editorial: MSC Communication in Physiological and Pathological Settings

**DOI:** 10.3389/fcell.2022.909550

**Published:** 2022-05-05

**Authors:** Philippe Bourin, Jeffrey M. Gimble, Louis Casteilla, António Salgado

**Affiliations:** ^1^ Independent Researcher, Toulouse, France; ^2^ Obatala Sciences Inc., New Orleans, LA, United States; ^3^ Institut RESTORE, Université de Toulouse, CNRS U-5070, EFS, ENVT, Inserm U1301, Toulouse, France; ^4^ Life and Health Sciences Research Institute (ICVS), School of Medicine, University of Minho, Braga, Portugal; ^5^ ICVS/3B’s Associate Lab, PT Government Associated Lab, Guimarães, Portugal

**Keywords:** mesenchymal stem cells, cell comunication, editorial, physiological settings, pathology

Mesenchymal stromal/stemcells (MSC), first identified within bonemarrow, have been studied since the late 1960s. Pioneering studies by Friedenstein and others showed that MSC had the potential for differentiation into osteoblasts, chondrocytes and adipocytes and that, in addition, they supported hematopoiesis. Subsequent work highlighted the presence of similar cells in multiple tissue sites and uncovered other signature MSC properties such as their immunomodulatory and antifibrotic activities. Furthermore, *in vivo* pre-clinical animal models and first-in-human clinical trials have extended these analyses of MSC to evaluate their transplantation actions in many types of disorders. The ability of MSC to communicate with their local microenvironment and systemically is now appreciated as their predominant mechanism of action, making them a privileged tool for regenerative medical applications, through mechanisms of action that are a direct consequence of their microenvironmental and/or systemic communication capacity. However, the means of communication can be varied according to the physiological or pathophysiological situation and may include the secretion of small molecules, cytokines, adipokines, microRNA, and exosome/vesicles as well as direct cell-cell interactions mediated via surface receptors. This special issue contains a series of articles over-viewing the current status of MSC’s communication avenues in the context of pathophysiological settings and clinical translation. These nine papers focus on two central themes (Planat-Benard et al., 2021): the crosstalk of MSC and immune cells and; (2) the involvement of MSC in tissue physiology and pathophysiology.

The first theme, immune modulation by MSC, is an emerging topic of discovery. Bazzoni et al. review the crosstalk of MSC with the immune cells via extracellular vesicles. They evaluate pleiotropic action of MSC relevant to the pathways involving both for innate and adaptative immune systems. Stevens et al. extend this theme with an in depth look at the crosstalk between MSC and the mononuclear phagocytic system. This manuscript presents multimodal data relating to paracrine secretion, metabolic reprogramming, organelle donation, extracellular vesicles and contact dependent communications. Planat et al., emphasizes that a common and central point between the MSC features of multipotency paracrine activity, and physical cell-cell interactions, especially with immune inflammatory cells, is the key importance of metabolism that governs their fate and behaviors. Finally, Kang et al. present experimental data showing that macrophages exert considerable influence on the differentiation of MSC in pathophysiological conditions. They report that in type 2 diabetes mellitus, macrophages at rest display an inflammatory phenotype and impaired bone regeneration.

Manuscripts within the second theme explore the role of MSC in various physiological or pathological conditions. Two manuscripts examine the interactions between the MSC and the other cells of the bone microenvironment. Arthur and Gronthos review the role of Eph-Ephrin system on bone physiology and hematopoiesis, showing that multiple Eph receptors and Ephrin ligands underpin MSC physiology, cartilage and bone homeostasis or hematopoiesis. In a related study, Takam Kamga et al. bring an interesting perspective to the role of the Notch and Wnt pathways as they relate to communication between MSC and hematopoietic cells in both healthy and leukemic subjects. They demonstrate how physiological signaling can be hijacked by cancer cells for their own protection at the expense of the neighboring MSC. Two experimental manuscripts provide support for a potential role of MSC to mitigate pathological conditions. Chu et al. show that amnion MSC can reduce the impact of hypoxia on trophoblasts. This effect is correlated with downmodulation of the mTOR pathway via EZH2 and hypothetically could be the mechanism of support for a clinical trial on the use of MSC therapy to mitigate or treat preeclampsia. Lu et al. explore the benefit of MSC conditioned medium to protect endothelium via a multifactorial mechanism involving both protein growth factors and small molecules such as carbon monoxide. Chiabotto et al. critically review the preclinical data showing that MSC impact liver fibrosis via mechanisms involving MSC engraftment and differentiation along the hepatic lineage as well as secretion of extracellular vesicles exerting anti-inflammatory and anti-fibrotic effects.

Together, this body of work provides a topical summary of the current status of our understanding of MSC communication in health and disease. We hope that these contributions will provide both insights and inspiration to the next generation of investigators exploring the fields of stromal/stem cell biology and regenerative medicine.
